# Thoracoscopic two-port treatment of two extralobar pulmonary sequestrations in the left thoracic cavity of a child: a case report

**DOI:** 10.3389/fped.2025.1570926

**Published:** 2025-04-25

**Authors:** Huashan Zhao, Shumin Zhao, Yunpeng Zhai, Rui Guo, Gang Shen, Hongxiu Xu, Sai Huang, Shisong Zhang

**Affiliations:** ^1^ Department of Thoracic and Oncological Surgery, Children’s Hospital Affiliated to Shandong University, Jinan, China; ^2^ Department of Thoracic and Oncological Surgery, Jinan Children’s Hospital, Jinan, China; ^3^ The Affiliated Central Hospital of Shandong First Medical University (Jinan Central Hospital), Jinan, China

**Keywords:** abdominal aorta, CT imaging, pediatric surgery, pulmonary sequestration, thoracoscopy, two-port technique

## Abstract

Reports of bronchopulmonary sequestration in two or more locations within the ipsilateral thorax in children are rare. To date, only a few clinical reports have described thoracoscopic two-port treatment for external lobe bronchopulmonary sequestration. We performed thoracoscopic two-port surgery on a 7-month-old girl and identified two abnormal masses in the left thoracic cavity: one located in the upper hilum of the lung and the other on the surface of the lower diaphragm. Both masses were resected using two-aperture thoracoscopic surgery and were pathologically confirmed as extralobar bronchopulmonary sequestrations. Preoperative computed tomography (CT) only detected the lesions in the upper hilum of the lung, indicating that CT alone is insufficient to achieve a comprehensive and accurate diagnosis of the disease in similar cases. Therefore, the thoracoscopic two-port technique can better diagnose and treat these diseases.

## 1 Introduction

Bronchopulmonary sequestration is a lung malformation in which diseased lung tissue is isolated and does not connect to the normal trachea or bronchus. It is classified as intralobar and extralobar pulmonary sequestrations according to anatomical features (1, 2). However, reports of extralobar pulmonary sequestrations in two or more locations within the same rib cage are rare. Published reports of extralobar pulmonary sequestrations in two locations within the same or both pleural cavities are mostly isolated cases (3–7). No cases of thoracoscopic two-port treatment for two locations of extralobar pulmonary sequestrations in the left thorax of children have been reported.

Therefore, we report a unique surgical case of a 7-month-old girl with two sites of extralobar pulmonary sequestrations in the left rib cage that were incidentally found during thoracoscopic two-port surgery. Both lesions were safely resected and pathologically confirmed as extralobar bronchopulmonary sequestration.

## 2 Case description

**Figure 1:**
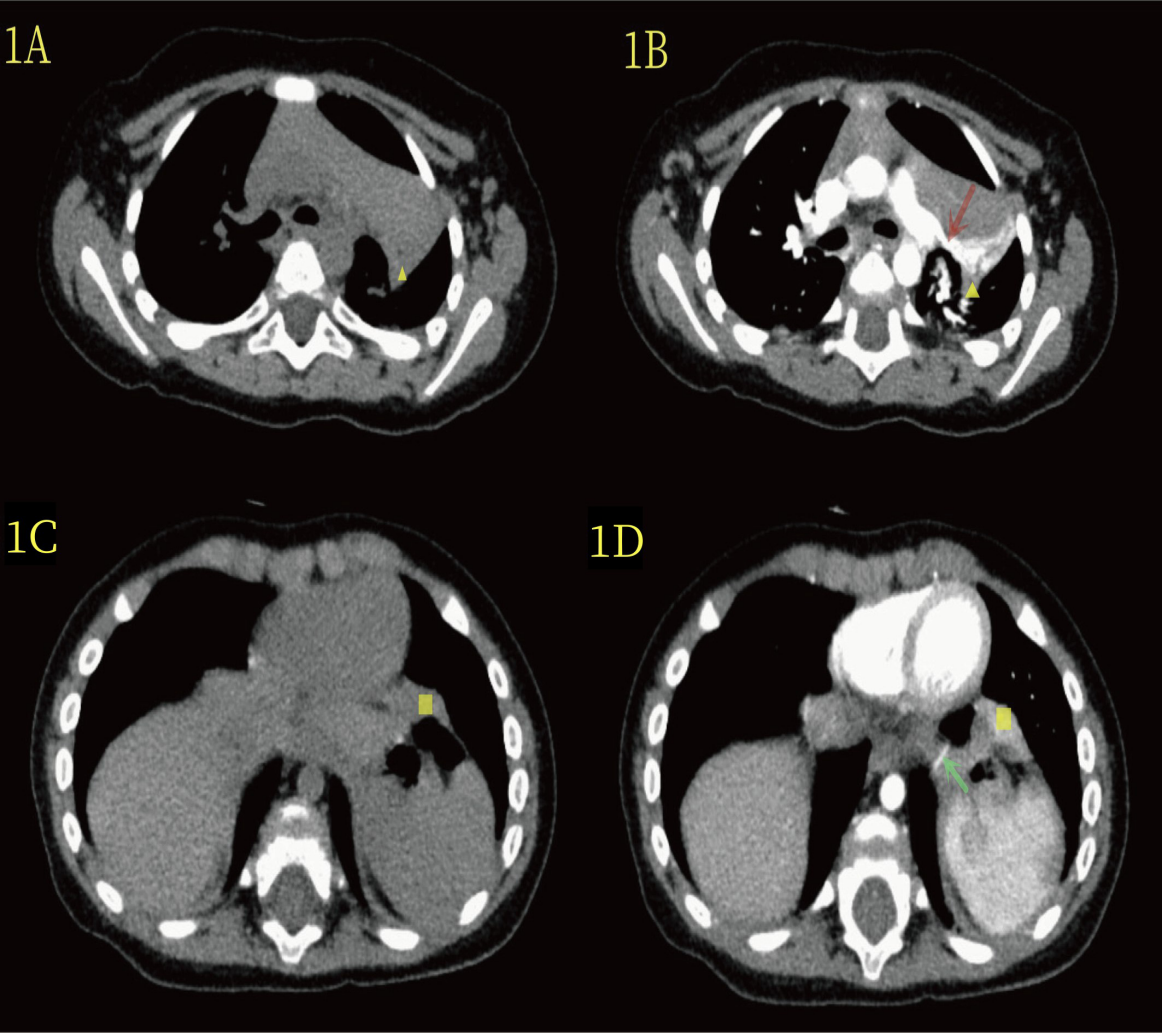
**(A)** The yellow triangle in the CT plain scan indicates the lesion at the upper hilum of the lung. **(B)** On enhanced CT scan, the yellow triangle highlights the lesion at the upper hilum of the lung, and the red arrow denotes the blood-supplying artery (branch of the upper pulmonary artery). **(C)** In the CT plain scan, the yellow quadrilateral indicates the lesion in the lower diaphragm. **(D)** On enhanced CT, the yellow quadrilateral marks the lesion in the lower diaphragm, and the green-clip head highlights the blood-supplying artery (abdominal aorta).

The patient was a 7-month-old girl diagnosed with a left thoracic lung malformation via ultrasound in the 24th week of her mother's pregnancy. A color ultrasound performed at delivery later indicated enlargement of the lesion. The child was born full term via vaginal delivery, without dyspnea or other discomfort. However, her parents were concerned that the malformation of the lungs might affect her respiratory function and physical development. She was referred to our hospital for a chest computed tomography (CT) scan, which revealed a clear, well-defined mass at the hilus of the lung in the left thoracic cavity (Figure 1A,B), with obvious enhancement in the arterial phase. The mass artery originated from the left pulmonary artery. Upon reevaluating the CT scan during thoracoscopic surgery, another mass was found on the surface of the diaphragm (Figure 1C,D). The mass artery in the diaphragm was small and originated from the abdominal aorta. Consequently, the parents were particularly worried about the risk of infection, bleeding from the lung disease or twisting of the lung tissue, and strongly requested hospitalization. She was admitted to the hospital, and relevant preoperative examinations were conducted, revealing no abnormalities or contraindications.

**Figure 2:**
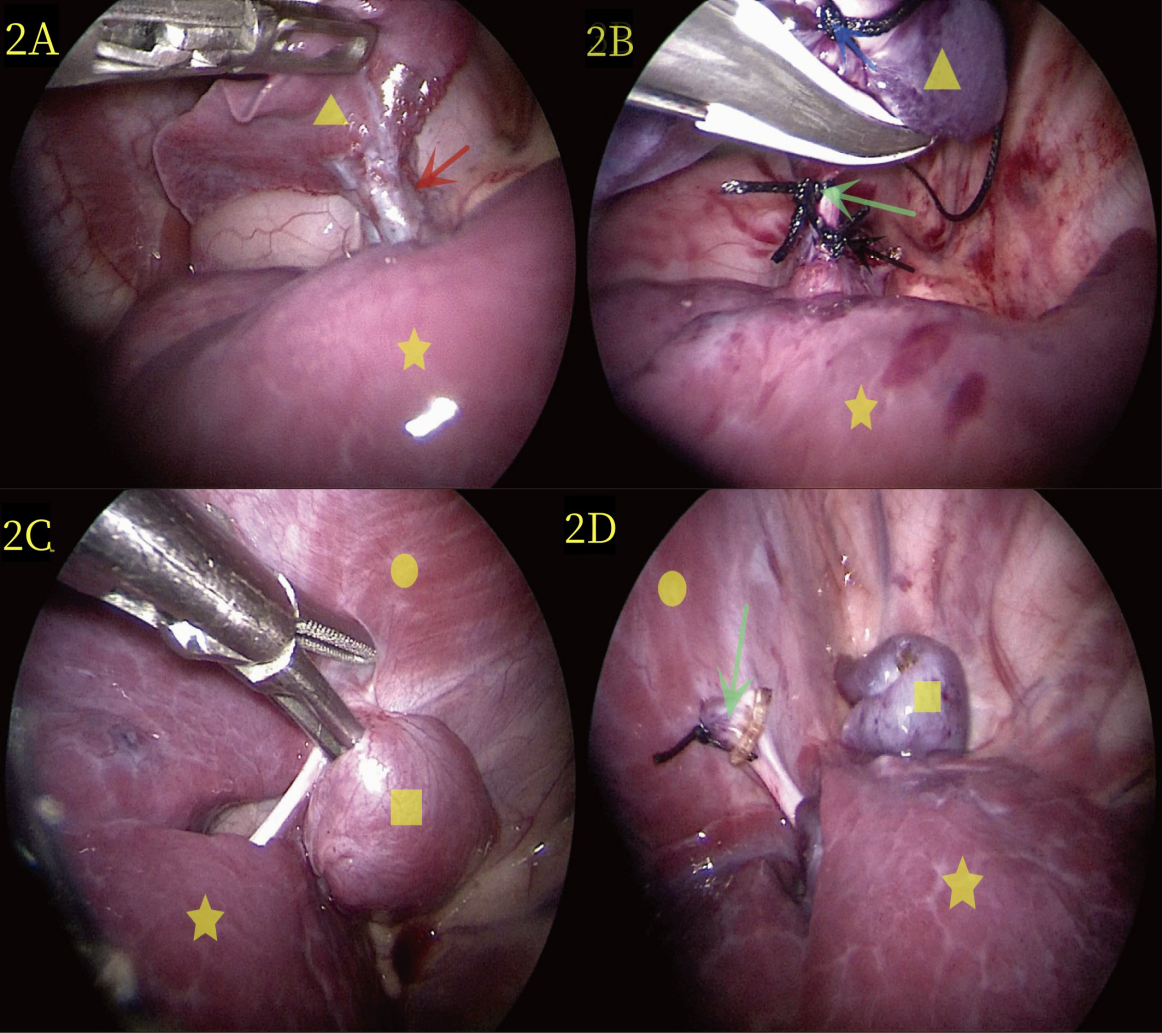
**(A)** During laparoscopic surgery, the yellow triangle represents the lesion at the upper hilum of the lung, the yellow five-pointed star denotes the normal lung, and the red arrow represents the vessel at the upper hilum of the lung. **(B)** Thoracoscopic surgery revealed extralobar pulmonary sequestration at the upper pulmonary portal (yellow triangle). Distal ligation and suspension (blue arrow), proximal ligation (black arrow), and suturing (green scissors) were performed, and images of resection were completed using LigaSure. **(C)** During thoracoscopic surgery, the yellow quadrilateral identifies the lesion in the lower diaphragm, the yellow circle represents the diaphragm, and the yellow five-pointed star marks the normal lung. **(D)** The lower diaphragm extralobar pulmonary sequestration lesions are revealed during thoracoscopic surgery (yellow quadrilateral); the green scissors showed the severed end of the blood vessel post-resection.

**Figure 3:**
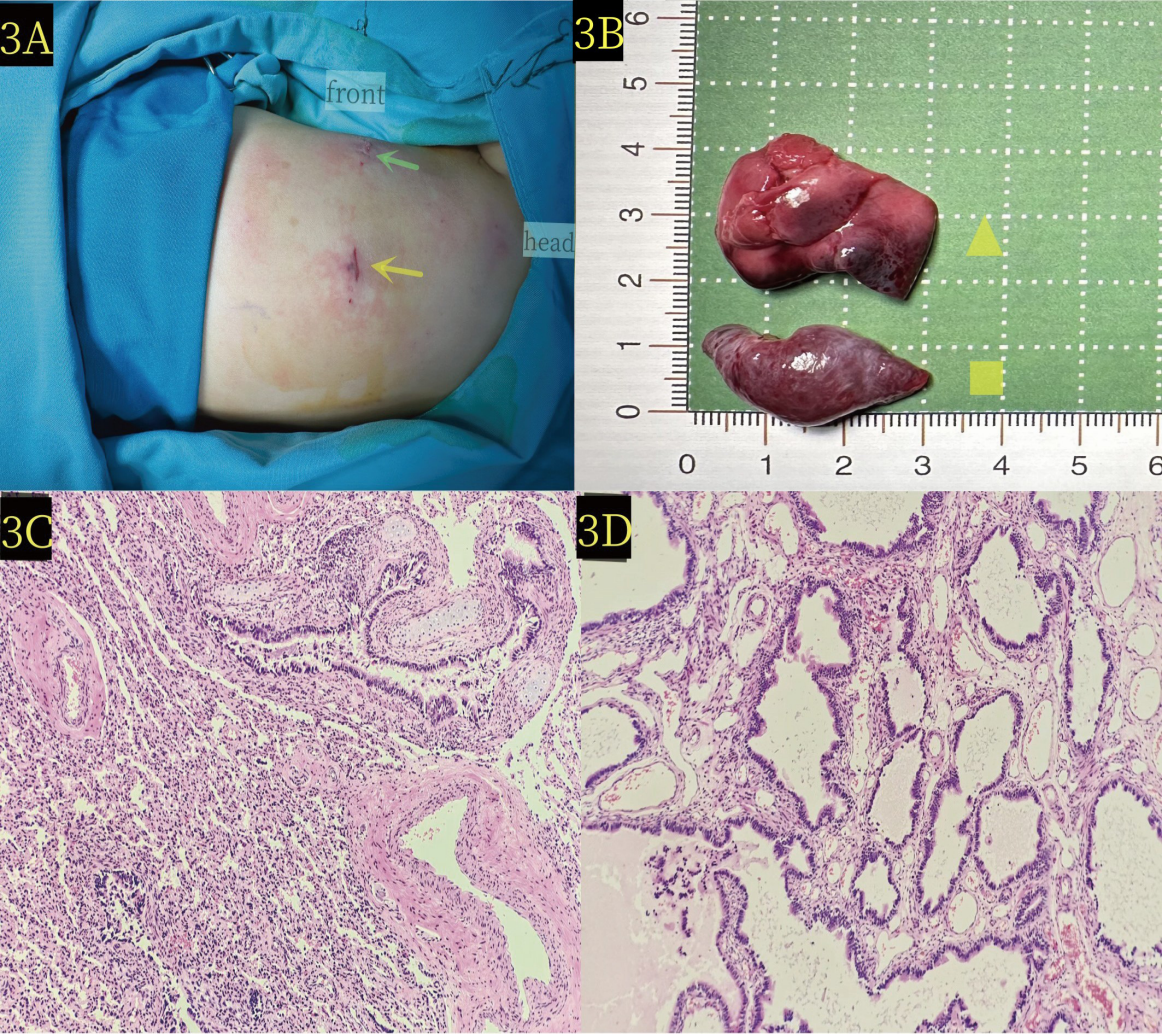
**(A)** The patient was positioned laterally on the right side during the operation. Green scissors and yellow arrows denote the locations of the thoracoscopic entry points. **(B)** Pathological image reveals the extralobar pulmonary sequestration lesion at the upper hilum of the lung (yellow triangle) and the lower diaphragm (yellow quadrilateral) x. **(C)** Microscopic pathology of the upper hilus pulmonis shows extralobar pulmonary sequestration lesion (hematoxylin and eosin stain, ×10). **(D)** Microscopic pathology of the lower diaphragm lesion confirms extralobar pulmonary sequestration in the (hematoxylin and eosin stain, ×10).

The patient underwent thoracoscopic two-port surgery on the third day after admission. Following general anesthesia, she was placed in a right lateral position (Figure 3A). A small intercostal incision, approximately 0.5 cm long, was made along the 7th posterior axillary line on the left side and was cut into the chest by layer. A 5-mm Trocar was inserted to establish a CO_2_ pneumothorax with a pressure of 4 mmHg and a flow rate of 2 L/min. After thoracoscopy with Trocar, a small incision of 0.5 cm was made at the 6th intercostal space of the left anterior axillary line, and a 5-mm Trocar was inserted. Thorax exploration revealed that the patient had two separate lumps that were far apart, with different surface colors. Therefore, we classified the two lesions as upper and lower lesions. The upper lesion was a dark red mass located near 3 cm × 3 cm × 2 cm of the hilum of the lung (Figure 2A). Additionally, the diseased vessels above connect to the hilum of the lung. The lower lesion is a bright red mass near 2 cm × 2 cm × 1 cm of the diaphragm (Figure 2C). It is solid, relatively free, and not visibly connected to the pulmonary lobe. Moreover, the diseased blood vessels below connect to the diaphragm.

The surgeon was positioned on the right side of the operating table, and the assistant sutured a 2-0 silk thread with a needle from the chest wall into the chest cavity. Next, the surgeon used an observer with one hand and an endoscopic operating instrument with the other to suspend the isolated lobar lung at the upper pulmonary hilum. The 2-0 silk thread with needle was reinserted and ligation was performed at the proximal end of the vascular pedicle, followed by suturing using the same 2-0 silk thread with needle. LigaSure was used to treat and cut the vascular pedicle (Figure 2B), and no bleeding was observed. Subsequently, the external lobar bronchopulmonary sequestration in the lower diaphragm was treated. The surgeon stood at the head of the child and successfully excised the lesion in the lower diaphragm using the same method (Figure 2D). Specifically, the operative time was 50 min, and the intraoperative bleeding was approximately 1 ml. The appearance of the two lesions removed showed that the lesions in the upper hilum of the lung (yellow triangle in Figure 3B) and the lower diaphragm (yellow quadrilateral in Figure 3B) were dark and bright red, respectively. Pathological observation with hematoxylin and eosin staining showed numerous malformed blood vessels in the lesions at the upper hilus of the lung, with visible bronchial structures containing cartilage (Figure 3C). The bronchial structures without cartilage were visible in the lesions of the lower diaphragm (Figure 3D).

The patient was discharged on postoperative day 3 with a total hospital stay of 5 days. Notably, neither chest tube drainage nor urinary catheterization was required postoperatively. Three-month follow-up examination revealed excellent recovery without any complications such as hemorrhage, pneumothorax, or infection. The patient remained free of any abnormal findings during both the immediate postoperative period and subsequent follow-up.

Supplementary Image 1 showcases a timeline with relevant data from the episode of care (Nursing roadmap).

## 3 Discussion

This is the first report regarding the treatment of two sites of extralobar pulmonary sequestrations in the left thoracic cavity using thoracoscopic two-port surgery. We found that the upper and lower diseased arteries originated from the pulmonary artery and abdominal aorta, respectively. Prior to this report, the rare cases of bronchopulmonary sequestration documented included multiple lesions in the unilateral thorax (5), multiple lesions in the bilateral thorax (3, 4, 6, 7), and anatomical variations in lesion location or blood vessels (8–10). Many of these rare cases were incidentally discovered during surgical exploration and confirmed to be rare lesions. Similarly, we report a case of extralobar pulmonary sequestrations, with one located in the hilus pulmonis that was diagnosed preoperatively and another found in the lower diaphragm during thoracoscopic two-port surgical exploration.

Bronchopulmonary sequestration occurs in approximately 0.15%–1.8% of the population, with extralobar pulmonary sequestrations comprising 15%–25% of these cases (1, 5). The lesion of extralobar pulmonary sequestrations has an independent visceral pleura, which is more clearly separated from the surrounding normal lung tissue. In the extralobar pulmonary sequestration lesions, 80% of the supply arteries originate from the thoracic or abdominal aorta, 15% from other systemic arteries, and 5% from the pulmonary artery (11–14). In the cases we reported, the two lesions were located in the left thoracic cavity, with the diseased arteries in the upper hilum of the lung and lower diaphragm originating from the pulmonary artery and abdominal aorta, respectively. This combination of arterial origin and the presence of both lesions is relatively rare. Extralobar pulmonary sequestration lesions occur 65% of the time between the left lung and the diaphragm (5, 10). Nowadays, with the improvement of prenatal ultrasound technology and the accumulation of physician experience, most fetal bronchopulmonary sequestration can be detected during pregnancy, and the lesion location can be determined (1, 5). In our reported cases, a single lesion in the left hilum of the lung was found by prenatal ultrasonography. Therefore, further imaging of the chest post-partum is required for diagnosis and treatment.

Imaging techniques for diagnosing bronchopulmonary sequestration include ultrasound, chest radiography, CT, and magnetic resonance imaging (MRI). Ultrasound and chest radiography can serve as preliminary screening tools (15, 16). Moreover, enhanced chest CT or MRI is crucial for confirming the diagnosis of bronchopulmonary sequestration (1, 5, 17, 18). Chest CT is widely used and clearly shows the location, size, and arteriovenous, among others (17, 18). However, in cases of extralobar bronchopulmonary sequestration, some of the arteries are thin and less visible on enhanced CT imaging, which can lead to the neglect of preoperative diagnosis (5). In the present case, the patient underwent preoperative enhanced chest CT, which showed an extralobar pulmonary sequestration lesion with a pulmonary artery supply at the left hilum of the lung, but no other lesion at the diaphragm. The management of extralobar pulmonary sequestration primarily focuses on the aberrant vessels. Preoperative contrast-enhanced CT scans were performed, with dynamic thin-section CT imaging employed to delineate the abnormal vasculature, as demonstrated in Figure 1A–D. While three-dimensional reconstruction was attempted, the image quality proved inferior to the dynamic thin-section CT due to technical limitations at our institution. When combined with the intraoperative findings, a review of the preoperative enhanced chest CT scan revealed a less obvious lesion in the diaphragm with a supply artery from the abdominal aorta. MRI has been shown to display vascular images in more detail and can also detect other small lung sequestration lesions that are not easily diagnosed by examination (1, 5). Therefore, preoperative MRI should be performed in children with bronchopulmonary sequestration.

Currently, controversies exist regarding the treatment of extralobar pulmonary sequestrations. Some researchers have suggested that patients with extralobar pulmonary sequestrations remain asymptomatic throughout their life or even experience spontaneous resolution (19, 20). Most studies indicate that patients with extralobar pulmonary sequestrations may have complications, such as infection, lesion torsion, and bleeding, which usually warrant surgical intervention (3, 5, 10, 21). In our case, extralobar pulmonary sequestrations were located in the hilum of the lung, which is prone to the risk of torsion. The parents of the child expressed a strong desire for surgery. A correct diagnosis of the disease was made through a thoracoscopic two-port surgery, and a successful surgical resection was performed. This approach eliminated the risk of lesion torsion and infection, and the parents of the child were pleased with the complete resection of the lesion and reduced surgical incisions. We used thoracoscopic two-port resection to completely resect two unconnected extralobar pulmonary sequestration lesions in the left thorax. This approach is novel, given the rarity of the disease. We hope our case report can serve as a reference for other healthcare professionals with similar cases. We implemented a modified two-port thoracoscopic approach as a technical improvement over the conventional three-port technique. By utilizing external suspension technology while maintaining surgical safety, this method achieved both port reduction and decreased consumption of surgical materials, resulting in better acceptance by both pediatric patients and their parents.

In conclusion, we report a case of extralobar pulmonary sequestrations in a 7-month-old child with two lesions located in the left thoracic cavity. The extralobar pulmonary sequestration lesion in the upper hilum and lower diaphragm supplied blood to the pulmonary artery and abdominal aorta, respectively. For such rare cases, enhanced chest CT alone cannot accurately diagnose all lesions. However, preoperative MRI can be added, and their combination can better diagnose the disease and help avoid preoperative diagnostic omission. The thoracoscopic two-port technique we used proved to be a safe and feasible method for diagnosing and treating this condition. This case highlights the importance of not overlooking imaging clues during preoperative examination and suggests that an MRI should be performed to assist in diagnosis if necessary. Moreover, careful exploration should be performed during surgery to avoid the omission of lesions.

## Data Availability

The raw data supporting the conclusions of this article will be made available by the authors, without undue reservation.
