# Supplementary figures and images for: Thoracoscopic two-port treatment of two extralobar pulmonary sequestrations in the left thoracic cavity of a child: a case report
Source: Front Pediatr. 2025 Apr 25;13:1570926. doi: 10.3389/fped.2025.1570926 (PMC12063352; doi:10.3389/fped.2025.1570926)

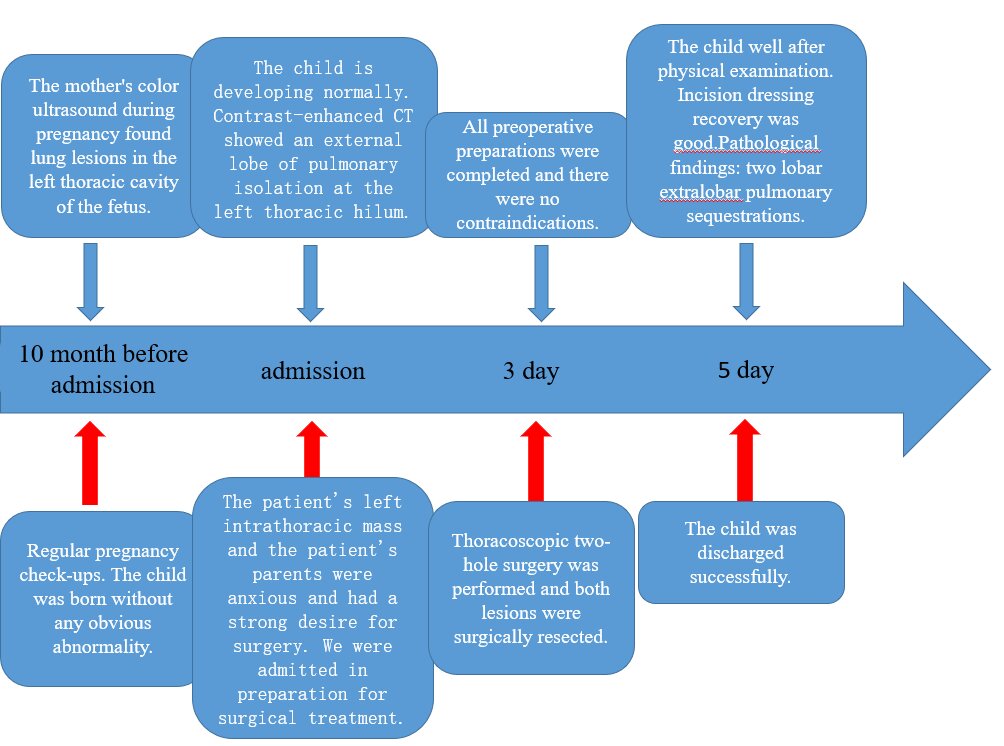

Supplement: Supplementary file 1 [file Image1.jpeg]
